# Overactivation of Notch1 Signaling Induces Ectopic Hair Cells in the Mouse Inner Ear in an Age-Dependent Manner

**DOI:** 10.1371/journal.pone.0034123

**Published:** 2012-03-20

**Authors:** Zhiyong Liu, Thomas Owen, Jie Fang, Jian Zuo

**Affiliations:** 1 Department of Developmental Neurobiology, St. Jude Children's Research Hospital, Memphis, Tennessee, United States of America; 2 Integrated Program in Biomedical Sciences, University of Tennessee Health Science Center, Memphis, Tennessee, United States of America; 3 University of Bath, Bath, United Kingdom; University of Otago, New Zealand

## Abstract

**Background:**

During mouse inner ear development, Notch1 signaling first specifies sensory progenitors, and subsequently controls progenitors to further differentiate into either hair cells (HCs) or supporting cells (SCs). Overactivation of NICD (Notch1 intracellular domain) at early embryonic stages leads to ectopic HC formation. However, it remains unclear whether such an effect can be elicited at later embryonic or postnatal stages, which has important implications in mouse HC regeneration by reactivation of Notch1 signaling.

**Methodology/Principal Findings:**

We performed comprehensive *in vivo* inducible overactivation of NICD at various developmental stages. In *CAG^CreER+^; Rosa26-NICD^loxp/+^* mice, tamoxifen treatment at embryonic day 10.5 (E10.5) generated ectopic HCs in the non-sensory regions in both utricle and cochlea, whereas ectopic HCs only appeared in the utricle when tamoxifen was given at E13. When tamoxifen was injected at postnatal day 0 (P0) and P1, no ectopic HCs were observed in either utricle or cochlea. Interestingly, Notch1 signaling induced new HCs in a non-cell-autonomous manner, because the new HCs did not express NICD. Adjacent to the new HCs were cells expressing the SC marker Sox10 (either NICD+ or NICD-negative).

**Conclusions/Significance:**

Our data demonstrate that the developmental stage determines responsiveness of embryonic otic precursors and neonatal non-sensory epithelial cells to NICD overactivation, and that Notch 1 signaling in the wild type, postnatal inner ear is not sufficient for generating new HCs. Thus, our genetic mouse model is suitable to test additional pathways that could synergistically interact with Notch1 pathway to produce HCs at postnatal ages.

## Introduction

The mouse inner ear is a well-organized sensory organ responsible for balance and hearing [1], [2], [3], [4]. It emanates from a thickening ectoderm adjacent to the hindbrain at approximately embryonic day 8 (E8), referred to as the otic placode, which continues to invaginate and morph into the otocyst [5], [6] at approximately E10. The otocyst is further patterned into the dorsal vestibular part of 3 cristae (for angular motion detection), utricle and sacculus (for linear motion detection), and the ventral part of coiled cochlea (for sound detection) [7]. Despite different morphologies, all parts contain sensory epithelia and adjacent non-sensory epithelia. Mechanosensory hair cells (HCs) and surrounding supporting cells (SCs) are located inside the sensory epithelium [2]. Mouse HCs and SCs are believed to have descended from the same prosensory progenitors, as seen in the case of the avian inner ear [8].

Current literature supports that Notch1 is the primary Notch receptor expressed in the mouse inner ear; thus, Notch1 signaling is thought to subserve all Notch activities during inner ear HC and SC development and to function via lateral induction and lateral inhibition [2], [9], [10], [11], [12], [13]. The lateral induction effect, mediated through Jagged1/Notch1 signaling, is involved in specifying the prosensory domains [14], [15], [16], [17], [18]. Consistent with this notion, loss of Notch1 causes formation of a smaller otic placode, whereas overactivation of NICD (Notch1 intracellular domain) in Pax2+ otic placode cells increases the size of the otic placode in *Pax2^Cre+^; Rosa26-NICD^loxp/+^* mice [19]. However, 2 recent reports show that Notch1 signaling might not be necessarily required to form the prosensory domain or maintain the properties of progenitor cells [20], [21]. Nonetheless, after the inner ear prosensory region is formed, prosensory progenitor cells undergo cell fate determination and become either HCs or SCs. Progenitors with high expression of Atoh1, a helix-loop-helix transcription factor required for HC formation, develop into HCs [22], [23], [24], [25], [26], [27], whereas those maintaining Notch1 signaling develop into SCs [9], [28]. Moreover, loss of or decreased Notch1 signaling in postnatal day 0 (P0) differentiating SCs after cell fate commitment can result in conversion of SCs into HCs [29], [30].

Overactivation of NICD in chick inner ears results in ectopic HCs [13], highlighting that Notch1 signaling is competent to specify the sensory domains that are permissive for further HC generation. Recently, *in vivo* overactivation of NICD at early embryonic stages has been shown to lead to ectopic HCs in mice [31], [32]. However, it remains unclear whether such an effect can also be elicited in late embryonic or postnatal stages *in vivo*. An insight into the effect of NICD overactivation at multiple time points at later ages will be informative to design future experiments that use overactivation of Notch1 signaling as a strategy for HC regeneration in mammals.

In this study, we induced constitutive overactivation of NICD in the developing mouse inner ear at various developmental stages. We demonstrated that overactivation of NICD at embryonic ages but not neonatal ages resulted in the formation of ectopic sensory epithelia (with HCs) in non-sensory regions and supernumerary HCs in sensory regions. Notably, new HCs did not express NICD, suggesting a non-cell-autonomous effect of Notch1 signaling. Taken together, our studies show that overactivation of NICD, in an age-dependent manner, can specify extra inner ear sensory patches or generate ectopic HCs. Our study also highlights the importance of the effects of developmental stages (or differentiation stages at the cellular level) on cellular reprogramming for regenerative purposes [33].

## Results

### Constitutive overactivation of NICD in otocyst cells generates ectopic hair cells (HCs) in the utricle

**Figure 1:**
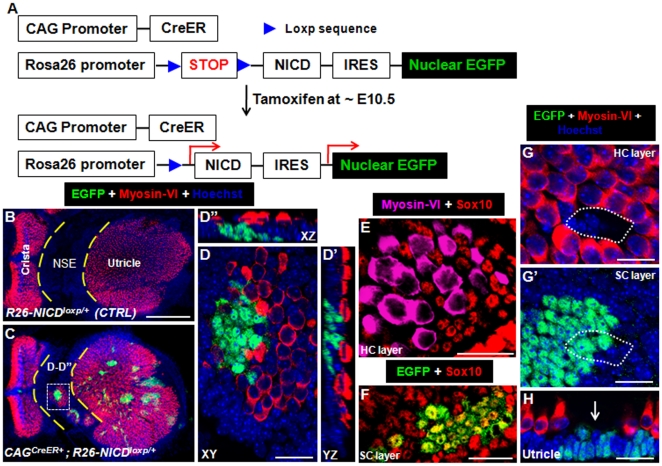
Ectopic hair cells (HCs) and supporting cells (SCs) in the utricle with overactivation of NICD at E10.5. (**A**) Diagram to illustrate the strategy of driving constitutive NICD expression. (**B–C**) Low-magnification image of the utricle of *Rosa26-NICD^loxp/+^* (B) and *CAG^CreER+^; Rosa26-NICD^loxp/+^* (C) embryos treated with tamoxifen at ∼E10.5 and analyzed at ∼E19. (**D–D″**) A high-magnification three-dimensional image of the white rectangular area marked in (C), which belongs to the utricle non-sensory region (NSE). (**E–F**) Triple staining of Myosin-VI, Sox10, and EGFP at the HC layer (F) and SC layer (G). (**G–H**) Whole mount (G–G′) and trans-section (H) images of the utricular endogenous sensory epithelium stained with EGFP and Myosin-VI. HCs were absent in the small white dotted line circled region in HC layer (G) and SC layer (G′). Arrow in (H) also points to the area where HCs are missing. NSE: non-sensory region; XY: Confocal XY plane; YZ: Confocal YZ plane; XZ: Confocal XZ plane. Scale bars: 200 µm in (B) and 20 µm in (D–H).

To activate NICD *in vivo* in cochlear cells at various developmental ages, we used *CAG^CreER+^; Rosa26-NICD^loxp/+^* mice that would be given tamoxifen at different developmental stages. NICD and EGFP cannot be expressed unless the preceding stop fragment is removed by Cre (Fig. 1A). After Cre-mediated recombination, the NICD will translocate into the nucleus and subsequently activate Notch1 signaling. As NICD and EGFP transcriptions are coupled by the same *Rosa26* promoter, EGFP will faithfully reflect NICD expression and also serve as a lineage tracer for EGFP/NICD expressing progenitors and offspring. Although the *CAG* promoter is ubiquitously active in most cases, with a single tamoxifen injection the mice can be considered mosaic, similar to the case of chimeric mice in which wild-type cells and mutant cells (with ectopic NICD) are randomly mixed together. Such a mosaic can be useful to analyze the lateral induction and lateral inhibition effects of Notch signaling.

*CAG^CreER+^; Rosa26-NICD^loxp/+^* embryos were given tamoxifen at ∼E10.5 and analyzed at ∼E19 (Fig. 1A). Large EGFP+ clusters were observed in both the utricles and cochleae. Although EGFP was also expressed in the cristae and sacculus, this study focused on only the utricles and cochleae. It should be noted that otocyst cells at ∼E10.5 targeted by tamoxifen were still rapidly proliferating. Thus, even though only a few cells were targeted, they could generate many EGFP+ descendants.

Neither EGFP+ cells nor ectopic HCs were found in the control samples (*n* = 3) of *Rosa26-NICD^loxp/+^* mice (Fig. 1B). However, in experimental utricle samples of *CAG^CreER+^; Rosa26-NICD^loxp/+^* mice (*n* = 4), ectopic EGFP+ sensory patches of different sizes were found in the non-sensory region between the utricle and cristae (Fig. 1C). This result indicates that single tamoxifen injection induces expression of NICD-IRES-EGFP in the otic precursor cells, and the EGFP+ cells have ectopic Notch signaling and become sensory progenitor cells. Inside each ectopic EGFP+ patch, there were always new HCs (40±11, *n* = 4) that were Myosin-VI+/EGFP−negative (Fig. 1D–D″). Interestingly, more ectopic HCs were present in larger EGFP+ patches. While the ectopic HCs were negative for the SC marker Sox10 (Fig. 1E), cells adjacent to the ectopic HCs expressed Sox10 [34]. These Sox10+ cells were either EGFP+ or EGFP−negative (Fig. 1F), both of which were defined as new SCs. Therefore, this result supports that the initial EGFP+ precursors, by lateral induction effect of Notch activities [13], [31], [32], specify additional progenitor cells that were EGFP−negative. Because of the constitutive NICD expression in the EGFP+ progenitors, they exclusively developed into EGFP+ SCs. The EGFP−negative progenitors differentiated into either new HCs or SCs, a process (to be discussed in detail later) that is reminiscent of the well-known lateral inhibition effect of Notch activities [16], [28], [35]. Such an explanation can be also applied to the following studies.

Inside the endogenous sensory epithelium of the utricle, most HCs (EGFP−) underwent normal differentiation (Fig. 1G). However, occasionally (in 2 out of 4 samples) HCs were absent (white dotted line circled region in Fig. 1G–G′ and arrow pointed area in Fig. 1H) in a very small contiguous area with only SCs (either EGFP+ or EGFP−negative).

### Constitutive overactivation of NICD in otic precursor cells generates new HCs in the cochlea

**Figure 2:**
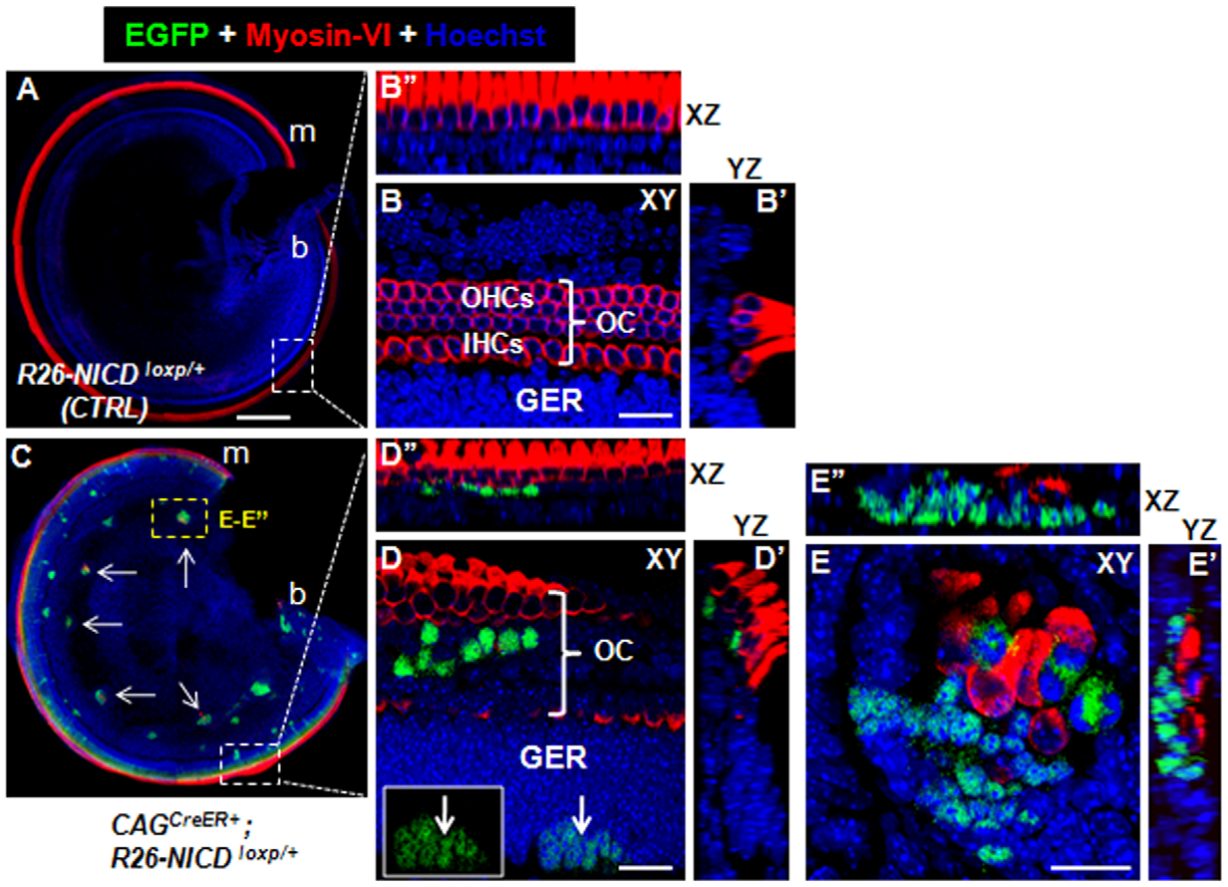
Ectopic HCs in the cochlea with constitutive Notch1 signaling at E10.5. (**A–B″**) Images of the cochlea taken from *Rosa26-NICD^loxp/+^* embryos treated with tamoxifen at ∼E10.5 and analyzed at ∼E19. (**C–E″**) Images of the cochlea taken from *CAG^CreER+^; Rosa26-NICD^loxp/+^* embryos treated with tamoxifen at ∼E10.5 and analyzed at ∼E19. White arrows in (C) point to ectopic sensory epithelia with new HCs in the spiral ganglion area. Inset in (D) shows the same EGFP+ patch (EGFP signal alone) visualized in the GER area in (D). (E–E″) High-magnification three-dimensional images of the yellow rectangular region in (**C**). OC: organ of Corti; GER: greater epithelium ridge; OHCs: outer hair cells; IHCs: inner hair cells; XY: Confocal XY plane; YZ: Confocal YZ plane; XZ: Confocal XZ plane. Scale bars: 200 µm in (A) and 20 µm in (B, D and E).

**Figure 3:**
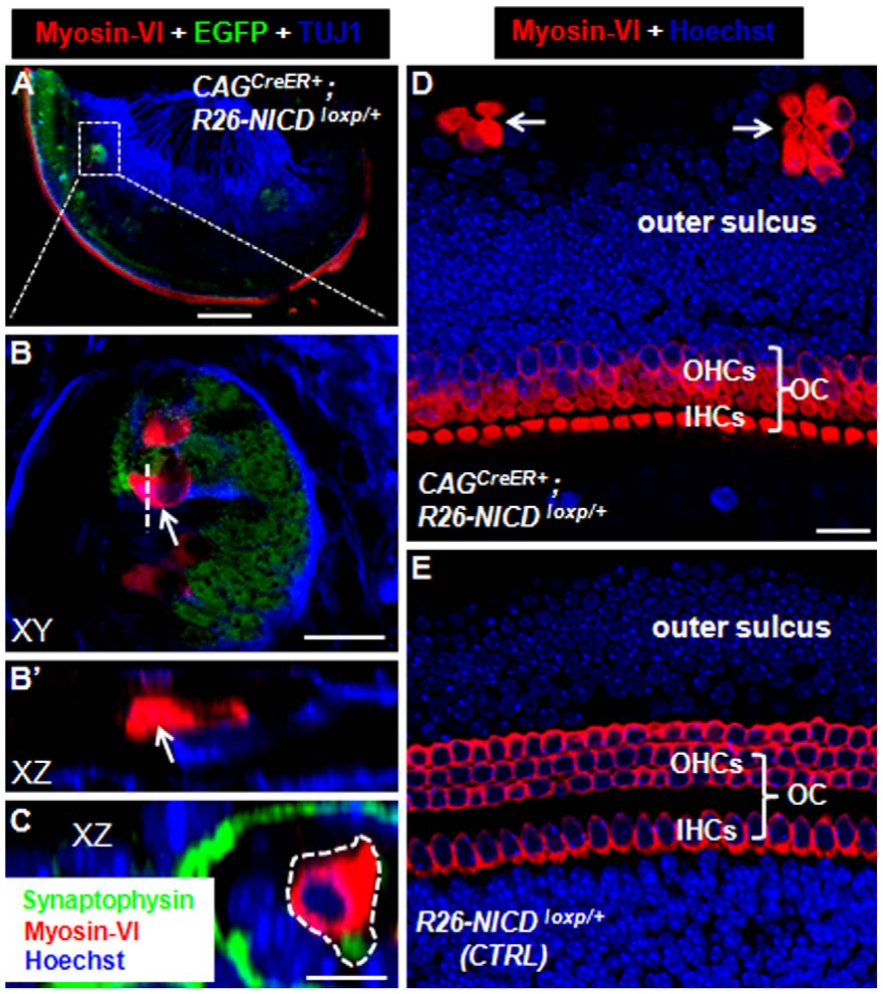
Ectopic HCs in cochlear spiral ganglion and outer sulcus region. (**A–B′**) Triple staining of TUJ1, Myosin-VI, and EGFP. (B) The high-magnification image of the square area in (A). (B′) The image of confocal XZ plane through the dashed line in (B). Arrows target the same ectopic HC in (B) and (B′). (**C**) Double staining image of Synaptophysin (a synaptic marker) and Myosin-VI in the cochlear spiral ganglion region. Very adjacent to the new HC (inside the dotted white circle) lies the Synaptophysin+ dot, suggesting presence of the synaptic structure. (**D–E**) Images of samples stained with Myosin-VI antibody in experimental (D) and control (E) cochlear samples. Ectopic HCs were present in outer sulcus regions in the experimental but not the control group. OC: organ of Corti; OHCs: outer hair cells; IHCs: inner hair cells; XY: Confocal XY plane; XZ: Confocal XZ plane. Scale bars: 200 µm in (A) and 20 µm in (B, D) and 10 µm (C).

In the ∼E19 control *Rosa26-NICD^loxp/+^* mice (*n* = 3), we found no ectopic HCs (Fig. 2A–B″). However, ectopic sensory areas containing Myosin-VI+/EGFP− HCs were also found in the cochlea of *CAG^CreER+^; Rosa26-NICD^loxp/+^* mice (*n* = 4) (Fig. 2C). Three regions had ectopic HCs in the cochlea. The first was the endogenous organ of Corti (Fig. 2D–D″). The expanded organ of Corti (in the medio-lateral dimension) with supernumerary HCs was found in the basal or apical turn. Notably, the greater epithelial ridge (GER) areas, which contained many EGFP+ cells, had no Myosin-VI+ HCs (inset in Fig. 2D). This observation was consistent with another recent study reporting that different chick inner ear cells respond differently to overactivation of Jagged1 [36]. The second region with ectopic HCs was the cochlear spiral ganglion (SGN) area (Fig. 2E–E″). Among the EGFP+ patches (15±5, *n* = 4), approximately half contained variable numbers (7±2) of new HCs. Interestingly, patches with new HCs were larger than those without new HCs. Furthermore, triple staining of Myosin-VI, EGFP, and Tuj1 demonstrated that the new HCs were distributed in the lateral edge of the SGN (Fig. 3A) and were innervated by neuronal fibers (Fig. 3B–B′). In addition, double staining of the synaptic marker Synaptophysin [37] and Myosin-VI supports the presence of the synaptic structure between the new HCs and neuronal cells in the SGN regions (white dotted line circled area in Fig. 3C). Of note, the Synaptophysin signal adjacent to new HCs was weaker than those around the neighboring neuronal cells, suggesting that the synaptic structures among new HCs and neuronal cells are not fully mature. The third region was the outer sulcus area in which ectopic clustered HCs (6±4, *n* = 4) were observed (Fig. 3D). In control samples, the outer sulcus area did not contain HCs (Fig. 3E).

**Figure 4:**
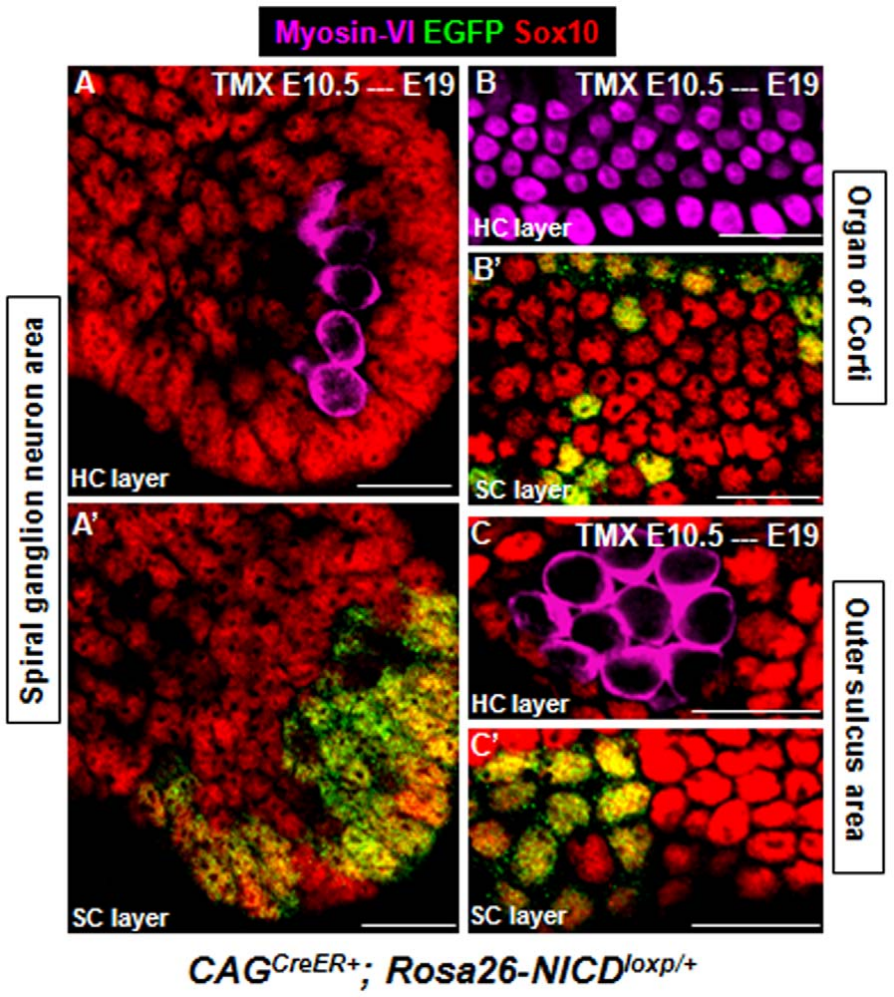
Expression pattern of Sox10 in ectopic cochlear SCs. Triple staining of Myosin-VI, Sox10, and EGFP of cochlear samples from *CAG^CreER+^; Rosa26-NICD^loxp/+^* embryos treated with tamoxifen at ∼E10.5 and analyzed at ∼E19. Ectopic HCs (Myosin-VI+) were Sox10− and SCs (EGFP+ or EGFP−) were Sox10+ in spiral ganglion neuron area (A–A′), organ of Corti (B–B′), and outer sulcus area (C–C′). Scale bars: 20 µm.

In all 3 areas, similar to what was observed in utricles (Fig. 1F–G), cells (either EGFP+ or EGFP−) surrounding these new HCs expressed Sox10 (Fig. 4), further supporting that ectopic HCs can drive the formation of ectopic SCs.

### New HCs appear in the utricle but not cochleae when ectopic NICD is turned on at ∼E13

**Figure 5:**
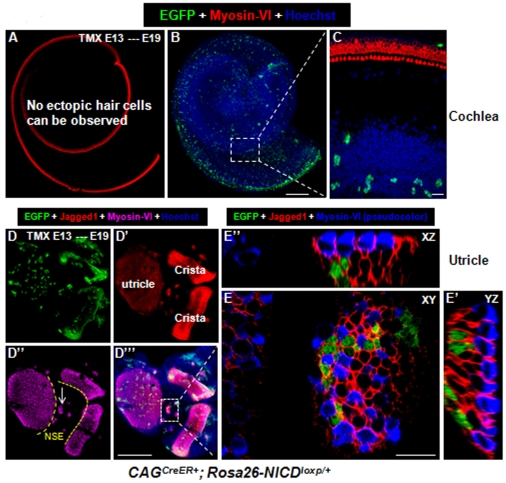
Overactivation of NICD at E13 generates new HCs in the utricle but not the cochlea. (**A–C**) Whole-mount cochlear image of a *CAG^CreER+^; Rosa26-NICD^loxp/+^* embryo treated with tamoxifen at ∼E13 and analyzed at ∼E19. Although many EGFP+ cells were present, no new HCs were observed. (**D–E″**) Whole-mount images of the utricle and 2 adjacent cristae from the same embryo. White arrows in (D″) point to the ectopic sensory epithelia region. (**E–E″**) A confocal three-dimensional, high-magnification image of the white rectangular region in (D′″). NSE: non-sensory region; XY: Confocal XY plane; YZ: Confocal YZ plane; XZ: Confocal XZ plane. Scale bars: 200 µm in (B and D′″) and 20 µm in (E).

Next, *CAG^CreER+^; Rosa26-NICD^loxp/+^* embryos were treated with tamoxifen once at ∼E13. Numerous EGFP+ cells were found in cochleae (*n* = 4), but no ectopic HCs were found at E19 (Fig. 5A–C). We further analyzed samples at E16 and E17 and did not find any new HCs (data not shown). Therefore, it is unlikely that new HCs first formed and subsequently died between E13 and E19, as further supported by the fact that new HCs generated by overactivation of Notch signals can survive until adult ages [32]. These results are consistent with a recent study reporting that no new HCs are present in cochlear explants (∼E13.5) transfected with NICD *in vitro* [20].

Although no ectopic HCs were found in *Rosa26-NICD^loxp/+^* control embryos (*n* = 3), variable numbers (16±6) of new HCs were found in most of the ectopic EGFP+ sensory patches (7±3, *n* = 4) present in each utricle (Fig. 5D–D′″). However, these EGFP+ patches were smaller than those found in *CAG^CreER+^; Rosa26-NICD^loxp/+^* embryos when tamoxifen was given at ∼E10.5 (Fig. 1). The ectopic sensory patches were distributed in the non-sensory region between the utricle and cristae (dashed line in Fig. 5D″).

**Figure 6:**
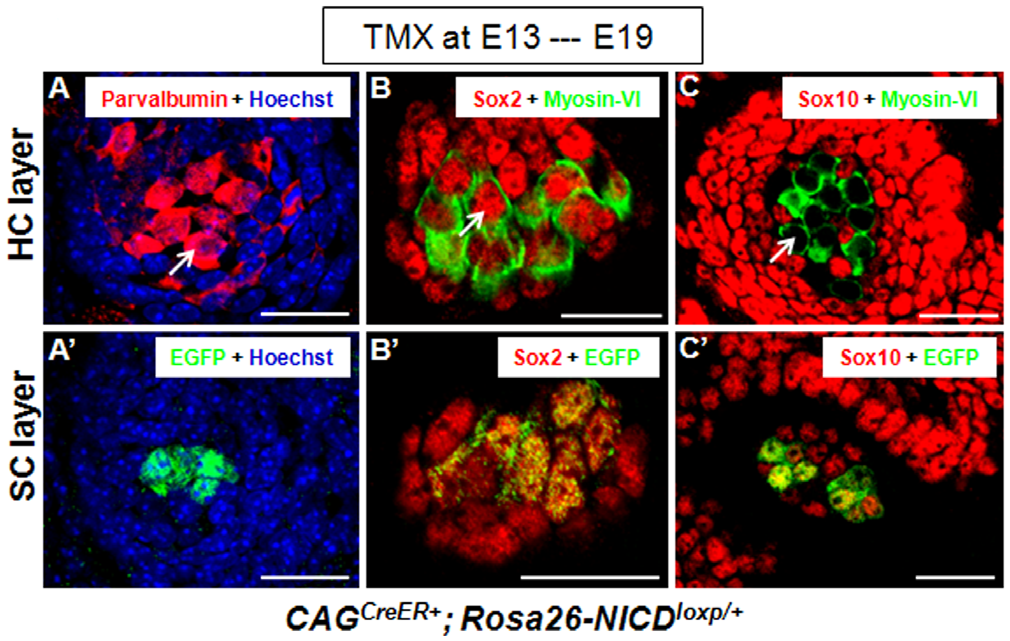
Expression of the sensory epithelium marker Parvalbumin and Sox2 in new HCs, and Sox10 in new SCs. (**A–A′**) Images of samples double stained with Parvalbumin and EGFP at HC layer (A) and SC layer (A′) of the ectopic sensory patches in the utricle non-sensory region of a *CAG^CreER+^; Rosa26-NICD^loxp/+^* embryo treated with tamoxifen at ∼E13 and analyzed at ∼E19. The arrow points to a new Parvalbumin+ HC. (**B–B′**) Triple staining of Myosin-VI, Sox2, and EGFP. Both ectopic HCs (B) and SCs (B′) were Sox2+. The arrow points to a new Sox2+/Myosin-VI+ HC. Of note, Myosin-VI was visualized in a pseudo-green color. (**C–C′**) Triple staining of Myosin-VI, Sox10, and EGFP. The arrow points to a new Myosin-VI+/Sox10−negative HC. The SCs (either EGFP+ or EGFP−negative) were Sox10+. Note that Myosin-VI was also visualized in a pseudo-green color. Scale bars: 20 µm.

Jagged1 is a sensory marker during early inner ear development [17], [31], [32]. It was clear that SCs (either EGFP+ or EGFP−) adjacent to the new HCs were Jagged1+ (Fig. 5E–E″). Because Jagged1 is a protein only expressed in the membrane, it was challenging to determine whether Jagged1 belonged to membranes of HCs or SCs wrapping the new HCs. Therefore, we additionally stained new HCs with another HC marker Parvalbumin [38]. Parvalbumin labeled the entire cell body of the new HCs (arrow in Fig. 6A) but did not label the SCs (Fig. 6A′). Furthermore, Sox2, another sensory marker [39], was expressed in both the new HCs (arrow in Fig. 6B) and SCs (either EGFP+ or EGFP−negative) (Fig. 6B′). Last, the new HCs were Sox10−negative (arrow in Fig. 6C) but the adjacent SCs (either EGFP+ or EGFP−negative) were Sox10+ (Fig. 6C′).

### Turning on ectopic NICD after birth fails to generate new HCs

**Figure 7:**
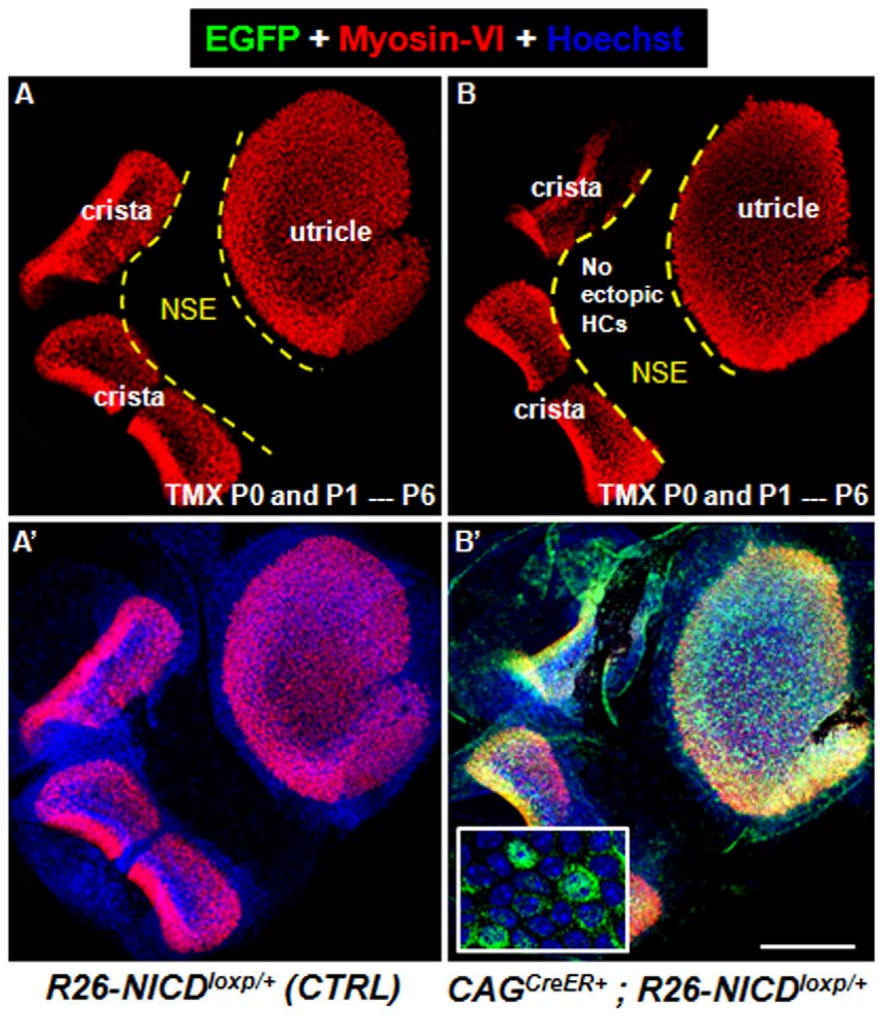
Overactivation of NICD in the postnatal utricle fails to generate new HCs at P6. Double staining of Myosin-VI and EGFP of utricles from *Rosa26-NICD^loxp/+^* control mice (A–A′) and *CAG^CreER+^; Rosa26-NICD^loxp/+^* experimental mice (B–B′). Both groups were treated with tamoxifen at P0/P1 and analyzed at P6. Although EGFP+ cells were present, no ectopic HCs were found in the experimental group. NSE: non-sensory region. Scale bars: 200 µm.

To determine whether non-sensory cells in the neonatal utricle still responded to NICD overactivation and generated ectopic HCs, *Rosa26-NICD^loxp/+^* (control group, *n* = 3) and *CAG^CreER+^; Rosa26-NICD^loxp/+^* (experimental group, *n* = 3) pups were given tamoxifen at P0 and P1, and analyzed at P6 (*n* = 3). No ectopic HCs were found in the non-sensory area of utricles from both control (Fig. 7A–A′) and experimental group (Fig. 7B–B′), although many EGFP+ cells were observed in experimental group (inset in Fig. 7B′). To test whether new HCs would emerge at older ages, we further analyzed *CAG^CreER+^; Rosa26-NICD^loxp/+^* at P10 (*n* = 3). No new HCs were found. However, the tracer EGFP became faint and difficult to visualize at P10, possibly because of decreased *Rosa26* promoter activity and efficiency of IRES-mediated EGFP translation. Similar difficulties have been encountered in studies on older retina cells in which *Rosa26-NICD^loxp/+^* mice were used [40].

**Figure 8:**
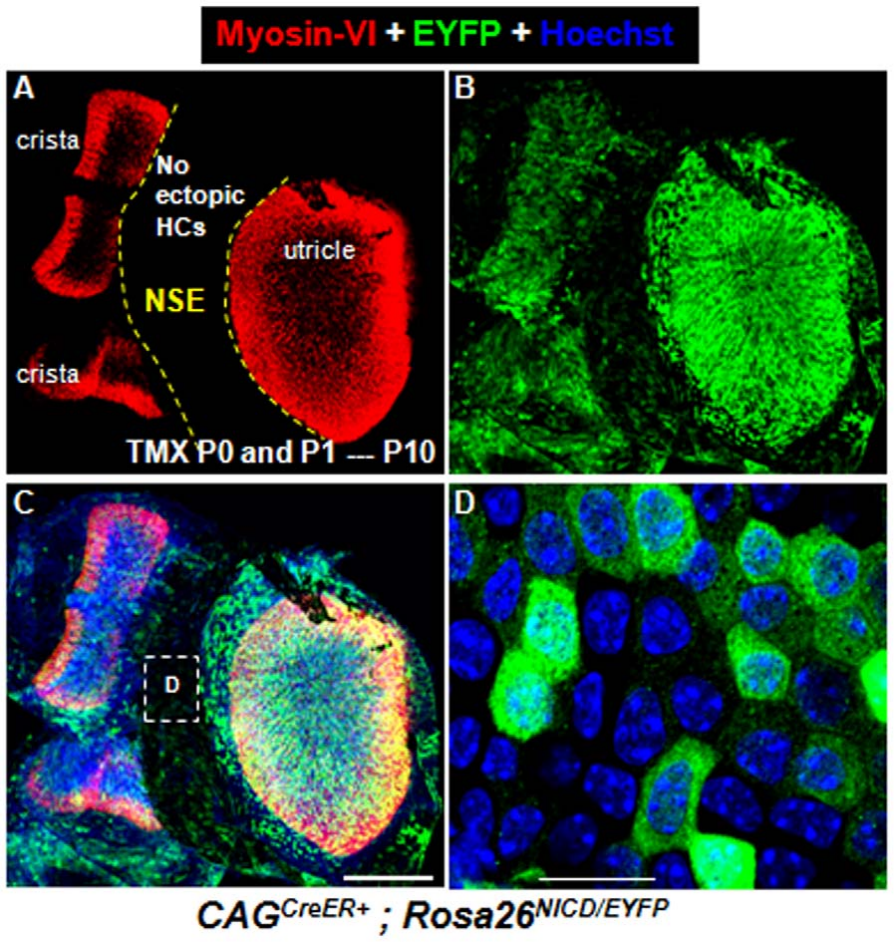
Overactivation of NICD in the postnatal utricle fails to generate new HCs at P10. (**A–C**) Double staining of Myosin-VI and EYFP of utricles dissected from *CAG^CreER+^; Rosa26^EYFP/NICD^* mice that were treated with tamoxifen at P0/P1 and analyzed at P10. (**D**) The high-magnification image of the squared area in (C). No ectopic HCs were present in the non-sensory area. NSE: non-sensory region. Scale bars: 200 µm in (C) and 20 µm in (D).

To better visualize cells with Cre-mediated recombination at P10, we crossed *CAG^CreER+^; Rosa26-NICD^loxp/loxp^* with *Rosa26-EYFP^loxp/loxp^* to get *CAG^CreER+^; Rosa26^EYFP/NICD^* mice as the experimental group, and *Rosa26^EYFP/NICD^* littermates were controls. EYFP was used to visualize cells with ectopic NICD. We could visualize EYFP but not EGFP, because EYFP is translated by the cap-dependent mechanism and EGFP by the IRES-mediated mechanism, which is known to have less translation efficiency. *CAG^CreER+^; Rosa26^EYFP/NICD^* mice were treated with tamoxifen at P0 and P1, and analyzed at P10 (Fig. 8). Many EYFP+ cells were observed, but again no new HCs were identified (Fig. 8). We could not analyze samples after P10 because of lethality. We speculated that mice died because of ectopic NICD in other organs [19].

## Discussion

We show that the potential of Notch1 signaling in specifying sensory patches is transient and declines with differentiation. Furthermore, cochlear cells become insensitive to ectopic NICD earlier than utricle cells. These findings are of particular interest for conducting future studies on HC regeneration in postnatal mammals. Also, it will be interesting to further test whether Notch overexpression could induce HCs under pathogenic conditions (i.e. ototoxic drug or noise-induced HC damage).

### Lateral induction and lateral inhibition effects of Notch1 signaling

Notch1 signaling elicits lateral induction effects at early embryonic ages when prosensory progenitors are being specified and lateral inhibition effects at later embryonic ages when HC and SC differentiation starts [2], [9], [10], [13], [16], [17], [28], [35]. The phenotypes observed in our *CAG^CreER+^; Rosa26-NICD^loxp/+^* model can be explained by these 2 sequential but different effects. EGFP and NICD share the same *cis*-transcription element, but NICD translation is cap dependent whereas EGFP translation is IRES dependent. The efficiency of IRES-mediated protein translation is generally lower than that of 5′ cap-dependent translation. This notion was further supported by the evidence that EGFP was hard to visualize in utricle samples of *CAG^CreER+^; Rosa26-NICD^loxp/+^* mice at P10, but EYFP was highly expressed in utricle samples of *CAG^CreER+^; Rosa26^EYFP/NICD^* mice (Fig. 8). It is certain that EGFP+ cells will be NICD+ populations, but the level of NICD in EGFP−negative cells (based on the sensitivity of the immunostaining technique) might still be lower than the threshold dosage required to effectively induce Notch1 signaling, as supported by previous studies involving this *Rosa26-NICD^loxp/+^* strain [19], [40], [41]. Thus, for the purpose of this discussion, we define NICD+ cells as EGFP+ cells and NICD−negative cells as EGFP−negative cells.

**Figure 9:**
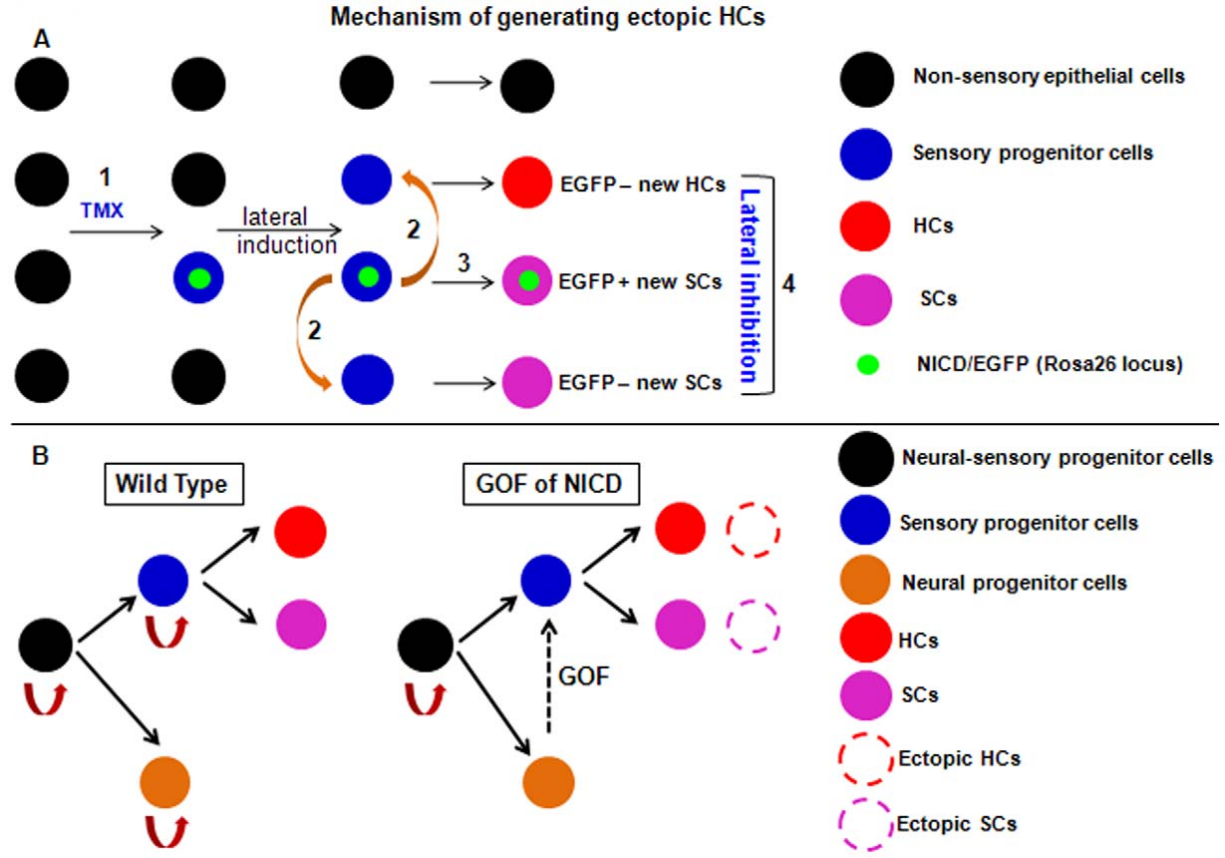
Working models to explain the generation of ectopic HCs and SCs in utricles and cochleae. (**A**) Non-sensory cells targeted by tamoxifen have ectopic NICD/EGFP expression (EGFP+). These EGFP+ cells become sensory progenitors (1). They also entitle neighboring cells (EGFP−negative) to be sensory progenitors by lateral induction (2). Next, ectopic EGFP+ sensory progenitors commit to EGFP+ SCs (3), whereas ectopic EGFP−negative sensory progenitors develop into either HCs or SCs (4). Lateral inhibition occurs between new EGFP−negative HCs and SCs, which might be mediated by Notch1 signaling from the endogenous Notch1 locus. (**B**) Gain of function (GOF) of NICD converts neural progenitor cells into sensory progenitor cells (dotted arrows), with generation of ectopic HCs and SCs (dotted circles) in the cochlear spiral ganglion area. TMX: tamoxifen.

Given the permissive cellular environment, NICD overactivation induced new HCs in the EGFP+ patches in both utricles and cochleae. All new HCs were EGFP−negative, and some SCs were EGFP+. Although the emergence of EGFP+ SCs can be easily explained by the prosensory-promoting ability of Notch1 signaling, new EGFP−negative HCs and SCs should be generated by the communication between EGFP+ cells and EGFP−negative cells, known as the lateral induction effects of Notch1 signaling (summary model in Fig. 9A). In other words, a single tamoxifen injection first transforms some non-sensory cells into prosensory progenitors (EGFP+). These EGFP+ prosensory progenitors in turn further trigger their neighboring non-sensory cells, which are not directly targeted by tamoxifen and are EGFP−negative, to turn on Jagged1 (Fig. 5) and Notch1 signaling, leading eventually to the transformation of these EGFP−negative cells into prosensory progenitors as well. Thus, with EGFP as a lineage tracer, new prosensory progenitors can be divided into EGFP+ and EGFP−negative groups. EGFP−negative progenitors can differentiate into HCs or SCs. We speculate that the final cell fate of each EGFP−negative progenitor is mediated by Notch1 signaling among different EGFP−negative progenitors (here NICD from the endogenous Notch1 but not Rosa26 locus might be involved), referred to as lateral inhibition between HCs and SCs. In contrast, EGFP+ progenitors are prevented from committing into HCs because they constitutively express NICD (at the Rosa26 locus), and hence EGFP+ progenitors exclusively develop into SCs. Last, because in the normal cochlear development HC differentiation precedes SC differentiation, and newly generated HCs can induce additional SCs [24], we propose that in our studies emergence of new HCs occurs earlier than new SCs as well. However, we currently lack the precise marker to define the SC fate and Sox10 is turned on in the entire otic vesicle epithelium [34], which prevents us from determining the onset of the new SC formation.

Inside the endogenous sensory epithelium of the utricle, HC density was sometimes slightly decreased in *CAG^CreER+^; Rosa26-NICD^loxp/+^* embryos injected with tamoxifen at ∼E10.5. This phenotype also can be explained by the lateral inhibition effect of Notch1 signaling. If ectopic NICD is induced in too many endogenous prosensory progenitors and subsequently not enough progenitors are left for HC commitment, the HC density will likely decrease. Such a pattern was occasionally found in endogenous sensory epithelium regions where EGFP+ clusters were extremely large (Fig. 1G–G′). In most endogenous sensory epithelium regions, if only a small fraction of progenitor cells has ectopic NICD expression (EGFP+), the overall progenitor pool might buffer the ectopic NICD and still assign enough sources for HC differentiation.

Of note, recently two reports showed that Notch signaling might not be required in specifying and/or maintaining the properties of the cochlear sensory progenitor cells in the early embryonic stages, because available sensory markers were expressed normally without Notch signaling [20], [21]. Given that Notch signaling becomes stronger (based on NICD antibody staining) in the later embryonic cochlear development [42], it is possible that other signaling pathways, such as Wnt signaling [19], could compensate the loss of Notch signaling during the early embryonic ages when overall Notch activities were slightly low.

### Roles of Notch1 signaling in development of the cochlear neuronal lineage

Mouse inner ear neural and sensory progenitors are believed to originate from the Ngn1+ neural-sensory progenitors [1]. This hypothesis, especially in the inner ear vestibular part, is further supported by the lineage tracing study with *Ngn1^CreER+^; Z/EG* mice [43] and another study reporting that deletion of *NeuroD1* leads to ectopic HCs in vestibular ganglia [44]. However, only cochlear SGNs and not cochlear prosensory progenitors were traced in *Ngn1^CreER+^; Z/EG* mice, raising the question of whether cochlear SGNs and prosensory progenitors derive from the same neural-sensory progenitors.

The observation that delaminating neuroblasts (neural progenitors) express the Notch ligand Delta1 [45] supports that sensory progenitors are gradually specified among the neural-sensory progenitors by Notch1 signaling. In our study, the presence of ectopic HCs in cochlear SGN regions of *CAG^CreER+^; Rosa26-NICD^loxp/+^* embryos (Figs. 2, 3 and 4) suggests that cochlear SGN progenitors with ectopic NICD are converted to prosensory progenitors, which can further differentiate into either HCs or SCs (summarized model in Fig. 9B), even though we cannot rule out the possibility that some of the new HCs might originate from glia cells. In addition, defective Notch1 signaling caused by deletion of *Delta1* resulted in expanded neural regions [16]. Taken together, our results support that cochlear sensory progenitors and SGN progenitors originate from identical neural-sensory progenitors. Because of the different *in vivo* and *in vitro* experimental conditions, or different levels of NICD, our results might not necessarily conflict with another recent report in which Notch signaling promotes cultured inner ear stem cells to follow neural lineage differentiation [46].

### Comparison of three different models explaining the induction of ectopic HCs in mice

Besides our *CAG^CreER+^; Rosa26-NICD^loxp/+^* model, two other mouse genetic models have been recently used to show that overactivation of NICD can induce the generation of ectopic HCs [31], [32]. These three models complement each other to provide consorted evidence of Notch1 signaling activities in the developing mouse inner ear. The advantage of the Pan et al. model [31] is that it combines the mouse Cre/loxP and Tet-On genetic system. NICD is transiently overactivated so that only lateral induction of Notch1 signaling is augmented and subsequent lateral inhibition is intact. Thus, ectopic prosensory progenitors can become either HCs or SCs. This model is different from our model in the following ways: 1) ectopic HCs can be found in cochlear SGNs regions across the entire turns in our model (Fig. 2) but exclusively in basal turns in the Pan et al. model; 2) ectopic HCs can be found in the cochlear ventral part in our model (Fig. 2) but in the dorsal part only in the Pan et al. model; 3) our data show that not all cells (∼E10.5) respond to NICD and become prosensory progenitors, but all cells seem to do so in the Pan et al. model. These differences may arise because different non-sensory cells were targeted: all types of cells were randomly targeted in our model, whereas *Col2a1Cre* activity determined the scope of the targeted cell population in the Pan et al. model.

The Hartman et al. model [32] is similar to our model in that NICD is constitutively overactivated. The tamoxifen-independent *hGFAPCre* bypassed the dystocia problem encountered in our model, thereby allowing the analysis of juvenile and adult inner ears. In the Hartman et al. model, the presence of ectopic HCs at adult ages suggests that they can survive for a long time and are possibly functional. However, *hGFAPCre* will overactivate NICD soon after Cre is active and this model therefore cannot be used to induce Notch1 signaling at various ages. Our tamoxifen-dependent *CAG^CreER+^* allowed the overactivation of NICD at E10.5, E13 and P0/P1, by which we were able to show that there is an age-dependant decrease in the responsiveness of inner ear non-sensory cells to Notch1 signaling.

### Implication of Notch1 signaling in HC regeneration in mammals

After HC damage, non-mammalian vertebrates (e.g., birds, fish and amphibians) can regenerate HCs [47] but mammals (e.g., mice and humans) either completely lose or have limited regenerative capacity in different inner ear sensory epithelia [3], [48], [49], [50]. Similar to the chick inner ear study [13], our study and 2 other reports [31], [32] show that either constitutive or transient overactivation of NICD can induce ectopic HCs in the mouse. These results can have significant implications on studies of HC regeneration after HC damage in mammals. The non-sensory cells adjacent to the endogenous sensory epithelium might be good candidates for manipulating Notch1 signaling.

Not all EGFP+ patches contain ectopic HCs and only embryonic non-sensory cells respond to NICD overactivation and generate new HCs, suggesting that other factors or signals besides Notch1 signaling may be needed to generate a bona fide sensory epithelium permissive for mechanosensory HC formation [51]. In support, Notch1 signaling has been shown to be required to maintain but not to initiate prosensory patch formation [18]. Although these factors have yet to be identified, previous studies have shown that during inner ear morphogenesis, Fgfr [11], [52], [53], [54], [55], [56], Wnt [57], [58], [59], BMP [60], [61], [62], [63], and Sonic Hedgehog (Shh) [63], [64], [65], [66] signaling pathways are involved in patterning the prosensory area. It is possible that a combined modulation of these signals triggers the conversion of postnatal non-sensory cells to HCs.

## Materials and Methods

### Mice

*Rosa26-EYFP^loxp/loxp^* (stock number: 006148) and *Rosa26-NICD^loxp/loxp^* (stock number: 008159) lines were purchased from The Jackson Laboratory (Bar Harbor, ME). The *CAG^CreER+^* mouse line was obtained from Dr. Guillermo Oliver, with permission of Dr. Andrew McMahon. Mice were crossed at 5 p.m. and the next morning was designated as E0.5 when vaginal plugs were found. Pregnant female mice were given tamoxifen (intraperitoneal, 100 µg/g body weight) [67] once when embryos were at ∼E10.5 and ∼E13. Neonatal mice were given tamoxifen (3 mg/40 g body weight) at P0 and P1.

### *In vivo* approach of overactivating of NICD in the mouse inner ear at different ages

*CAG^CreER+^; Rosa26-NICD^loxp/+^* mice were given tamoxifen at different developmental stages. In the previous study where *CAG^CreER+^; Rosa26-LacZ^loxp/+^* embryos received single tamoxifen treatment at early embryonic ages [68], detectable recombination event (X-gal+ cells as read-out) occurred within 6 hours and became more apparent within 24 hours after tamoxifen treatment. Because we were using the same *CAG^CreER+^* mouse line and the *NICD-IRES-EGFP* is also knocked into the same *Rosa26* locus, we reasoned the timing of turning on ectopic NICD (or Notch1 signaling) should be 0.5 or 1 day after ∼E10.5 tamoxifen injection and this should be applicable to other tamoxifen injection time points as well in our study.

Moreover, the level of NICD overexpression is dictated by the *Rosa26* promoter, which is moderate active and about 1/8 −1/10 of the activities of the *CAG* promoter [69], [70], as it would otherwise result in nonphysiological responses such as cell death. In addition, all progenitors and their progeny that express EGFP after tamoxifen induction should maintain the *Rosa26* promoter–driven ectopic NICD expression permanently, so that their effects can be studied *in vivo* until the time of analysis. Which cells are induced by tamoxifen injection is largely determined by the CAG promoter of *CAG^CreER+^* and the tamoxifen dose [68].

In this study, we gave the earliest tamoxifen injection at ∼E10.5, because it was difficult to get live mutant embryos at perinatal ages when the pregnant mother was given tamoxifen at ∼E9.5 or earlier. Because tamoxifen frequently caused dystocia, we analyzed embryos at ∼E19 (equivalent to P0).

### Ethics Statement

All animal work conducted for this study was approved by the Institutional Animal Care and Use Committee at St. Jude Children's Research Hospital and performed according to NIH guidelines.

### Histology and immunofluorescence

Samples of the inner ear were processed by our routine protocols described previously [71], [72]. All images were examined using a Zeiss LSM 700 confocal microscope. The following primary antibodies were used: anti-Myosin-VI (rabbit, 1∶200, 25-6791, Proteus Bioscience, Ramona, CA), anti-TUJ1 (mouse, 1∶1,000, MMS-435P, Covance, Princeton, NJ), anti-GFP (rabbit, 1∶50, A-21311, Invitrogen, Carlsbad, CA) or anti-GFP (chicken, 1∶1000, ab13970, Abcam, Cambridge, UK), anti-Parvalbumin (mouse, 1∶1000, P3088, Sigma), anti-Synaptophysin (mouse, 1∶200, 101011, Synaptic Systems), anti-Jagged1 (goat, 1∶300, sc-6011, Santa Cruz Biotechnology), anti-Sox10 (goat, 1∶250, sc-17342, Santa Cruz Biotechnology) and anti-Sox2 (goat, 1∶1000, sc-17320, Santa Cruz Biotechnology). The following secondary antibodies were used: donkey anti- rabbit Alexa Fluor 647 (1∶1000, A31573, Invitrogen), donkey anti-chicken DyLt 488 (1∶200, 703-486-155, Jackson ImmunoResearch, West Grove, PA), donkey anti-goat Alexa Fluor 568 (1∶1000, A11057, Invitrogen), goat anti-rabbit Alexa Fluor 568 (1∶1000, A11036, Invitrogen), goat anti-mouse Alexa Fluor 647 (1∶1000, A21236, Invitrogen) and goat anti-chicken Alexa Fluor 488 (1∶1000, A11039, Invitrogen). All images were taken with confocal microscope (Zeiss 700 model). Utricle and cochlear samples were scanned at 1 µm intervals.
